# A cross-sectional study on factors associated with hypertension and genetic polymorphisms of renin-angiotensin-aldosterone system in Chinese hui pilgrims to hajj

**DOI:** 10.1186/s12889-019-7357-1

**Published:** 2019-09-04

**Authors:** Yinxia Zhang, Fangfang Shi, Zhanbiao Yu, Aimin Yang, Maolan Zeng, Jiaoyue Wang, Haiping Yin, Benzhong Zhang, Xiao Ma

**Affiliations:** 1 West China School of Public Health and West China Fourth Hospital, Sichuan University, Chengdu, 610000 China; 2 Northwest Minzu University, Lanzhou, 730030 China; 3 Center for Disease Control and Prevention, Kongtong District, Pingliang, Gansu China; 4 Qingyang People’s Hospital, Qingyang, 745000 China; 5 Hong Kong Institute of Diabetes and Obesity, The Chinese University of Hong Kong, Kowloon, Hong Kong SAR; 6 Gansu International Travel Healthcare Center, Lanzhou, 730000 China; 7 School of Public Health, Lanzhou University, Lanzhou, 730000 China

**Keywords:** Pilgrims, Hypertension, RAAS, Polymorphism

## Abstract

**Background:**

Hypertension is the leading risk factor for cardiovascular disease (CVD), however, the studies on lifestyle and genetic risks in Chinese pilgrims to Hajj was limited. The aim of this study is to examine the prevalence and associated lifestyle and genetic risks for hypertension among Hui Hajj pilgrims in China.

**Methods:**

We performed a cross-sectional analysis of data in 1,465 participants aged 30–70 years who participated in a medical examination for Hui *Hajj* pilgrims from Gansu province, China in 2017. Multiple logistic regression was used to evaluate the association of potential risk factors with hypertension. Deoxyribonucleic acid (DNA) polymorphism was examined at sites in the renin-angiotensin-aldosterone system (*RAAS*).

**Results:**

The prevalence of hypertension was 47% among this population. Lifestyle factors such as fried food preference (like vs. dislike: odds ratio [OR]: =1.53, 95% confidence interval [CI]: 1.13–2.09) and barbecued food preference (like vs. dislike: OR = 1.45, 95% CI: 1.06–1.97) were associated with elevated risk of hypertension among Hui pilgrims. Comparing with Angiotensin converting enzyme (*ACE*) rs4425 *AA* genotype, *TT* genotype was associated with hypertension risk (OR = 2.16, 95% CI: 1.17–4.00). Similar results were also observed for *ACE* rs4437 *CC* genotype (OR = 1.95, 95% CI: 1.07–3.55), Angiotensin II receptor (*ATR*) rs129876 *AA* genotype (OR = 4.10, 95% CI: 2.30–7.32) and Aldosterone synthase (*CYP11B2*) rs1912 *TT* genotype (OR = 2.82, 95% CI: 1.57–5.06) genotypes.

**Conclusions:**

Unhealthy lifestyle and genetic factors were associated with the prevalence of hypertension in Chinese Hui pilgrims and their interactions were also observed.

**Electronic supplementary material:**

The online version of this article (10.1186/s12889-019-7357-1) contains supplementary material, which is available to authorized users.

## Background

Hypertension is an important risk factor of morbidity and mortality related to cardiovascular diseases, stroke and other diseases, which is related to both genetic and environmental factors [1]; The effects of hypertension on the body are slow-acting, heterogeneous, and varying among different ethnic groups [2, 3], particularly for those who are 65 years and older [4]. According to data from the World Health Organization, more than 1.5 billion people globally will have hypertension by 2025 [5]. Once in a lifetime every Muslim is expected to undergo a holy pilgrimage, known as Hajj, which takes place in the 12th month of the Islamic lunar calendar. Every year an estimated two million Muslim pilgrims gather from all around the world to perform the holy pilgrimage. Over the past few years cardiovascular diseases have emerged as an important cause both of intensive care unit (ICU) admission and of mortality during Hajj [6]. The burden of hypertension and its complications are substantial among Muslims from all over the world who make the Hajj pilgrimage to Mecca, Saudi Arabia [7, 8]. However, few studies have addressed the risk factors of hypertension on Muslim pilgrims till now.

More than 150 genes have been linked to hypertension and genetic risk factors appear to vary with time and study population [9, 10], the genes encoding the proteins involved in the renin-angiotensin-aldosterone system (*RAAS*) consistently appear to be key factors in hypertension. Blood pressure rises when renin and angiotensin-converting enzyme (*ACE*) convert angiotensinogen (*AGT*) into angiotensin I (*Ang I*) and angiotensin ll (*Ang II*). Then Ang II combines with its cognate receptor (angiotensin II receptor, *ATR*) [1, 11, 12]. Blood pressure is also directly regulated by aldosterone, which is produced by aldosterone synthase (*CYP11B2*)[13]. The susceptibility of these genes may play fundamental roles in the development of hypertension and therefore become the focus of our study.

In addition to genetic risk factors, lifestyle and behavioral factors also contribute to essential hypertension. These factors included over weight and obesity, alcohol drinking, tobacco smoking, physical inactivity, unhealthy dietary habits (adequate consumption of fruits and vegetables), having diabetes, elevated non-HDL cholesterol, and chronic kidney disease [14–16]. Though Muslims adhere to Islam’s prohibition against alcohol and smoking, [17] reports of Gansu Muslims who have made the Hajj pilgrimage indicated high prevalence of hypertension of 60.69% in 2013 [18] and always higher than the national average according to the Gansu International Travel Healthcare Center statistics. Few studies have addressed on Hajj pilgrims that are special ethnic group in China.

With the aim of understanding what genetic and lifestyle factors are responsible for the increased risk of hypertension among Hui Hajj pilgrims in China, we examined the hypertension prevalence and factors associated with hypertension as well as explored polymorphism in RAAS genes and lifestyle factors in Hui Hajj pilgrims.

## Methods

### Participants

We conducted a cross-sectional analysis based on data obtained from the medical examination for Hajj pilgrims from Gansu, China in 2017. We choose the entire participants who completed the medical examination for Hajj pilgrims. A total of 1,465 Hui pilgrims including 833 men and 632 women were contained with the mean age of 57.0 ± 9.4 years. All participants completed a demographic characteristic-related questionnaire, physical examination, and clinical tests, and participated in the Hajj pilgrimage to Mecca in 2017. Individuals were interviewed and subjected to a physical examination. Their blood samples were also collected in order to further conduct genetic testing and analysis. The study protocol was approved by the Medical Research Ethics Committee of the Northwestern University for Nationalities, and all participants provided written informed consent before enrollment.

### Data collection

Several types of data were collected in this study including questionnaire data obtained from in-person interviews, clinical data from physical examination and laboratory tests, and biospecimen collection. In-person interviews were conducted by trained interviewers using a standardized and structured questionnaire. The interviewers were trained to administer the questionnaire in a standardized fashion. Demographic information included age, sex, marital status, education, occupation, consumption of alcohol, use of tobacco, family history of hypertension, and dietary habits. Clinical data were obtained from physical examination and laboratory tests. According to the results of physical examination and the diagnostic criteria for hypertension, the subjects were divided into normal blood pressure group and hypertension group according to blood pressure values. The 200 biological samples were randomly selected from the each groups separately subjects for genetic polymorphism detection of RAAS system. A total of 357 qualified serum samples was included for DNA extraction, including 195 and 162 participants without and hypertension.

### Physical examination

The examination included a measurement of weight, height and blood pressure. Automatical recording instruments (DHM-301, DINGHENG, China) were used to measure weight and height. Body mass index (BMI) was calculated as weight in kilograms divided by the square of height in meters. Systolic blood pressure (SBP) and diastolic blood pressure (DBP) were measured by a well-trained physician, which was measured after the participants had been seated for 5-min. Individuals underwent three readings of arterial pressures in a seated position using an automatic pulse sphygmomanometer [19](UDEX-TWIN, Japan). Those with average systolic blood pressure ≥ 160 mmHg were re-measured three times the next morning. Laboratory tests included blood samples from all participants under fasting conditions in the morning at the International Health Center for Entry-Exit Health in Gansu Province. Routine laboratory tests were conducted including blood tests (Xeitomex XT-1800 Analyzer), blood biochemistry (Kerman Coulter AU680 Biochemistry Analyzer), electrocardiography (Japan Photoelectric ECG-9130P), and chest X-ray (DRX-1).

### Definitions

Based on diagnostic criteria of the World Health Organization and the International Society of Hypertension, hypertension was defined as systolic blood pressure ≥ 140 mmHg or diastolic blood pressure ≥ 90 mmHg or using anti-hypertensive therapies [20]. Diabetes was defined as fasting plasma glucose ≥126 mg/dL (≥7.0 mmol/L) or use of anti-diabetic medication at the time of the baseline interview (ADA, 2014). Tobacco smoking was defined as those who smoked at least one cigarette per day in the past 6 months. Alcohol drinking was defined as those who drank hard liquor, beer, or wine at least one time per week in the past 6 months. Family history of hypertension was based on participants reporting as having at least one parent, sibling, or offspring with hypertension.

### Genotyping of RAAS polymorphism

Genomic Deoxyribonucleic acid (DNA) was extracted from blood using a commercial kit (Omega Bio-Tek, D6919-01B). DNA concentrations were determined on a 1% agarose gel imaging system (Bio-RAD, USA) and a UV spectrophotometer (Pultton P200, USA). Samples (20 μL) were randomly selected from the qualified DNA extracts and diluted to 18–22 ng/μL for PCR amplification. The remaining samples were stored at − 80 °C for later use.

Polymerase Chain Reaction (PCR) was carried out to amplify regions within the coding sequences of the genes for *AGT* (accession: chromosome 1, NC_000001.11, gene ID: 183), *ACE* (chromosome 17, NC_000017.11, gene ID: 1636), *ATR* (chromosome 3, NC_000003.12, gene ID: 545), and *CYP11B2* (chromosome 8, NC_000008.11, gene ID: 1585). PCR primers were designed using Primer Premier 5.0 and Oligo 7 (Additional file 1: Table S1), then synthesized by Shanghai Biological Engineering (Shanghai, China). PCR reactions (25 μL) contained 1 μL of genomic DNA (18–22 ng/μL), 1 μL each of upstream and downstream primers (10 pmol/μL), 12.5 μL of 2X Taq PCR Master Mix (containing Taq DNA polymerase Mg^2+^•dNTPs), and 9.5 μL of sterilized ultrapure water.

PCR products were checked by agarose gel electrophoresis, then sent for sequencing to the Suzhou Jinweizhi Biological Technology Co., Ltd. Sequencing results were analyzed using DNAstar and DNAMAN software.

Finally, genotypes at polymorphic sites in *RAAS* genes were analyzed using high-resolution melting analysis. Primers were designed using Light Scanner software (Additional file 1: Table S2). To ensure accurate genotyping, the primers were tested on samples previously collected from individuals similar to the study population [21, 22]. In addition, melting curves were calibrated using high- and low-temperature internal standard (Additional file 1: Table S3) [23].

PCR reactions for high-resolution melting (11 μL) contained 1 μL of genomic DNA (18–22 ng/μL), 1 μL each of upstream and downstream primers (10 pmol/μL), 5 μL of 2X Taq PCR Master Mix (BioTeke, Beijing, China), 1 μL of LC Green saturated dye (Idaho Technology, USA) and 2 μL of ultrapure water. Reaction conditions were as follows: initial denaturation at 95 °C for 5 min; 35 cycles of initial denaturation at 94 °C for 20 s, renaturation for 20 s at the appropriate annealing temperature, and extension at 72 °C for 20 s; a final extension at 72 °C for 10 min; and storage at 4 °C. Then 1 μL each of the diluted high- and low-temperature internal standards were added to the reaction wells and incubated in a 95 °C water bath for 30 s, followed by incubation in a 25 °C water bath for 30 s. Signals were analyzed using a Light Scanner 96 high-resolution melting curve analysis system (Idaho Technology, USA).

### Statistical analysis

Descriptive statistics of continuous variables were obtained by calculating mean value and standard deviation (SD), and categorical variables were obtained by calculating the number and frequency distribution. T test or Chi-square test were used to detect the differences among participants’ characteristics. Furthermore, multiple logistic regression models were used to evaluate the association between hypertension and risk factors. Age were categorized into equally spaced categories: ≤40 years, 41–50 years, 51–60 years, and > 60 years. Years of education were categorized into meaningful spaced categories: ≤6 years (primary school), 7–9 years (middle school), 10–12 years (high school), > 12 years (college or above). Consistency of polymorphic genotypes with the predictions of Hardy-Weinberg equilibrium was tested using SHESIS software. Genotype frequencies were compared between hypertension and normal groups. All analyses were performed using IBM SPSS 20.0 Software (IBM, Chicago, IL, USA).

## Results

### Basic characteristics

The mean age of participants was 57 ± 9.4 years (57.7 ± 9.5 years for men, women: 56.1 ± 9.2 years for women), and the average BMI was 25.8 ± 3.4 kg/m^2^. 56.9% of the participants were male, and 46.1% of them were illiteracy. Most of the participants never smoked (92.6%) nor drank (95.9%) in their lifetime. The prevalence of hypertension was 47% among this population. The basic characteristics of participants overall and according to hypertension in those Chinese Hui pilgrims to *Hajj* were summarized in Table 1. Compared to participants without hypertension, those with hypertension were older, officer/technician, diabetics, and tended to have higher weight, Body mass index (BMI) and Fasting plasma glucose (FPG).

**Table 1:** Characteristics of Chinese pilgrims to *Hajj* by hypertension (*n* = 1465)

Characteristic	Hypertension			*P* value
	Normal	Cases	Overall	
Number of participants	777 (53.0)	688 (47.0)	1465 (100)	
Age (years)	54.5 ± 9.2	59.8 ± 8.8	57 ± 9.4	< 0.01
≤ 40	43 (5.5)	10 (1.5)	53 (3.6)	< 0.01
41–50	226 (29.1)	95 (13.8)	321 (21.9)	
51–60	311 (40.0)	247 (35.9)	558 (38.1)	
> 60	197 (25.4)	336 (48.8)	533 (36.4)	
Sex				
Male	425 (54.7)	408 (59.3)	833 (56.9)	0.08
Female	352 (45.3)	280 (40.7)	632 (43.1)	
Marital status^ƚ^				0.56
Married	774 (99.6)	686 (99.7)	1460 (99.7)	
Others	3 (0.4)	2 (0.3)	5 (0.3)	
Education (years)				
≤ 6	339 (43.6)	336 (48.8)	675 (46.1)	0.10
7–9	256 (32.9)	198 (28.8)	454 (31)	
10–12	110 (14.2)	82 (11.9)	192 (13.1)	
> 12	72 (9.3)	72 (10.5)	144 (9.8)	
Occupation				
Officer/technician	60 (7.7)	79 (11.5)	139 (9.5)	< 0.01
Tradesman	120 (15.4)	63 (9.2)	183 (12.5)	
Farmer	405 (52.1)	353 (51.3)	758 (51.7)	
Others	192 (24.7)	193 (28.1)	385 (26.3)	
Family income per month (CNY)				
< 2500	267 (34.4)	257 (37.4)	524 (35.8)	0.15
2500–3499	168 (21.6)	147 (21.4)	315 (21.5)	
3500–4500	68 (8.8)	75 (10.9)	143 (9.8)	
> 4500	274 (35.3)	209 (30.4)	483 (33.0)	
Height (cm)	162.5 ± 7.9	162.2 ± 8	162.4 ± 7.9	0.45
Weight (kg)	67.3 ± 11.3	69 ± 11.4	68.1 ± 11.4	< 0.01
SBP (mmHg)	126.6 ± 10.2	150.3 ± 8.9	137.7 ± 15.2	< 0.01
DBP (mmHg)	73.3 ± 13.3	79.4 ± 18.9	76.2 ± 16.4	< 0.01
FPG (mmol/L)	6.8 ± 1.4	7.2 ± 1.6	7 ± 1.5	< 0.01
BMI (kg/m^2^)	25.4 ± 3.4	26.1 ± 3.3	25.8 ± 3.4	< 0.01
Underweight (< 18.5)	11 (1.4)	4 (0.6)	15 (1)	< 0.01
Normal (18.5–24.99)	345 (44.4)	250 (36.3)	595 (40.6)	
Overweight (25–29.9)	353 (45.4)	356 (51.7)	709 (48.4)	
Obese (≥30)	68 (8.8)	78 (11.3)	146 (10.0)	
Tobacco smoking	55 (7.1)	53 (7.7)	108 (7.4)	0.65
Alcohol drinking	32 (4.1)	28 (4.1)	60 (4.1)	0.96
Fried food preference				
Dislike	230 (29.6)	210 (30.5)	440 (30)	0.75
Neither like nor dislike	175 (22.5)	162 (23.5)	337 (23)	
Like	372 (47.9)	316 (45.9)	688 (47)	
Barbecued food preference				
Dislike	331 (42.6)	279 (40.6)	610 (41.6)	0.12
Neither like nor dislike	224 (28.8)	179 (26)	403 (27.5)	
Like	222 (28.6)	230 (33.4)	452 (30.9)	
Worship frequency (days/week)				
< 3	174 (22.4)	133 (19.3)	307 (21.0)	0.34
3–5	179 (23)	169 (24.6)	348 (23.8)	
> 5	424 (54.6)	386 (56.1)	810 (55.3)	
Diabetes	260 (33.5)	312 (45.3)	572 (39.0)	< 0.01
Family history of hypertension	37 (4.8)	35 (5.1)	72 (4.9)	0.77

*Note.* ^*^ *SBP* and *DBP* systolic and diastolic blood pressure, *FPG* fasting plasma glucose, *BMI* body mass index. Data were presented as mean (SD) or n (%); mean ± SD: *t*-test; n (%): *x*^2^-test. ^ƚ^ Fisher’s exact test

### Lifestyle risk factors and hypertension risk

**Fig. 1:**
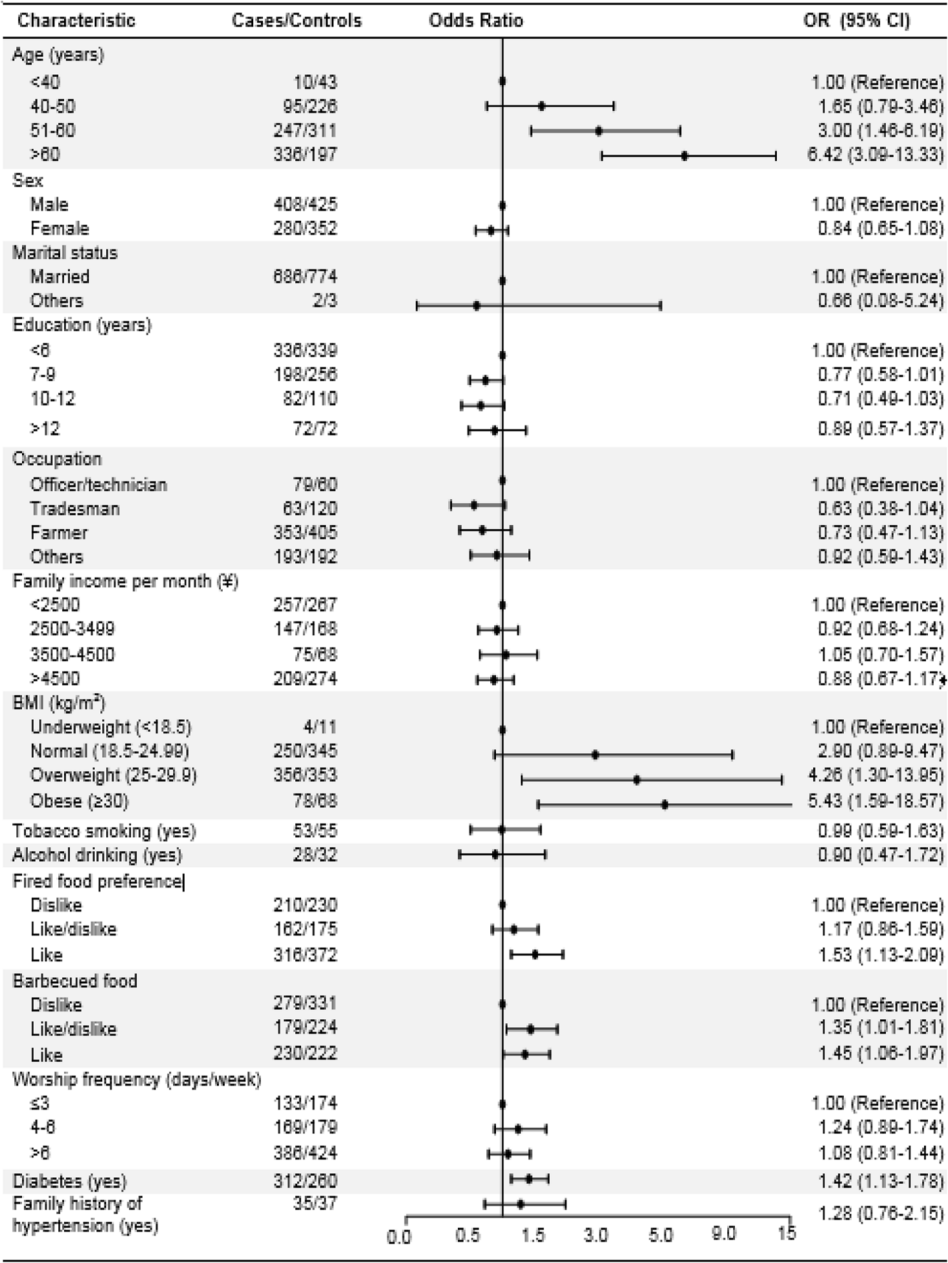
Associations between basic characteristics and hypertension risk in Hui pilgrims to *Hajj*. The dots and horizontal lines are ORs and 95% confidence intervals (CIs) using logistic regression analysis

Figure 1 showed the associations of lifestyle risk factors with hypertension risk among Hui pilgrims to *Hajj*. Age, BMI, barbecued food preference, fried food preference, and diabetes were significantly associated with higher odds of hypertension after adjusting for multiple confounding factors. Participants who like fried food had 53% increased risk of hypertension compared with those dislike fried food (odds ration [OR]: 1.53, 95% confidence interval [CI]:1.13–2.09). Compared to those with disfavor barbecued food, those with barbecued food preference were significantly associated with hypertension risk (OR: 1.45, 95% CI: 1.06–1.97).

### Genetic risk factors and hypertension risk

To further explore the role of genetic risk factors in the development of hypertension among Chinese pilgrims to *Hajj*, 357 qualified blood samples were randomly selected to conduct genetic testing. We controlled some of the factors for controlling major potential confounders. Table 2 presented the comparison of basic characteristics between participants with and without genetic tests. The result showed that there are no differences in participant’s characteristic between the two groups. For example, the percentage of overweight among participants with and without genetic tests was 44.8 and 49.5%, respectively (*P* = 0.31).

**Table 2:** Comparison of Characteristics between Participants with and without Genetic Tests

Characteristic	Participants		*P* value^*^
	Without Gene Tests	With Gene Tests	
Number of participants	1108 (75.6)	357 (24.4)	
Age (years)			
< 40	40 (3.6)	13 (3.6)	0.50
40–50	240 (21.7)	81 (22.7)	
51–60	434 (39.2)	124 (34.7)	
> 60	394 (35.6)	139 (38.9)	
Sex			
Male	629 (56.8)	204 (57.1)	0.90
Female	479 (43.2)	153 (42.9)	
Marital status			
Married	1104 (99.6)	356 (99.7)	0.82
Others	4 (0.4)	1 (0.3)	
Family income per month (Ұ)			
< 2500	411 (37.1)	113 (31.7)	0.06
2500–3499	245 (22.1)	70 (19.6)	
3500–4500	100 (9.0)	43 (12.0)	
> 4500	352 (31.8)	131 (36.7)	
BMI (kg/m2)			
Underweight (< 18.5)	11 (1.0)	4 (1.1)	0.31
Normal (18.5–24.99)	435 (39.3)	160 (44.8)	
Overweight (25–29.9)	549 (49.5)	160 (44.8)	
Obese (≥30)	113 (10.2)	33 (9.2)	
Tobacco smoking			
Yes	77 (6.9)	31 (8.7)	0.28
No	1031 (93.1)	326 (91.3)	
Alcohol drinking			
Yes	43 (3.9)	17 (4.8)	0.47
No	1065 (96.1)	340 (95.2)	
Worship frequency (days/week)			
< 3	240 (21.7)	67 (18.8)	0.10
3–5	273 (24.6)	75 (21.0)	
> 5	595 (53.7)	215 (60.2)	
Diabetes			
Yes	438 (39.5)	134 (37.5)	0.50
No	670 (60.5)	223 (62.5)	
Family history of hypertension			
Yes	48 (4.3)	24 (6.7)	0.07
No	1060 (95.7)	333 (93.3)	

*Note.* ^*^ The results from the *Chi Square Test*

**Fig. 2:**
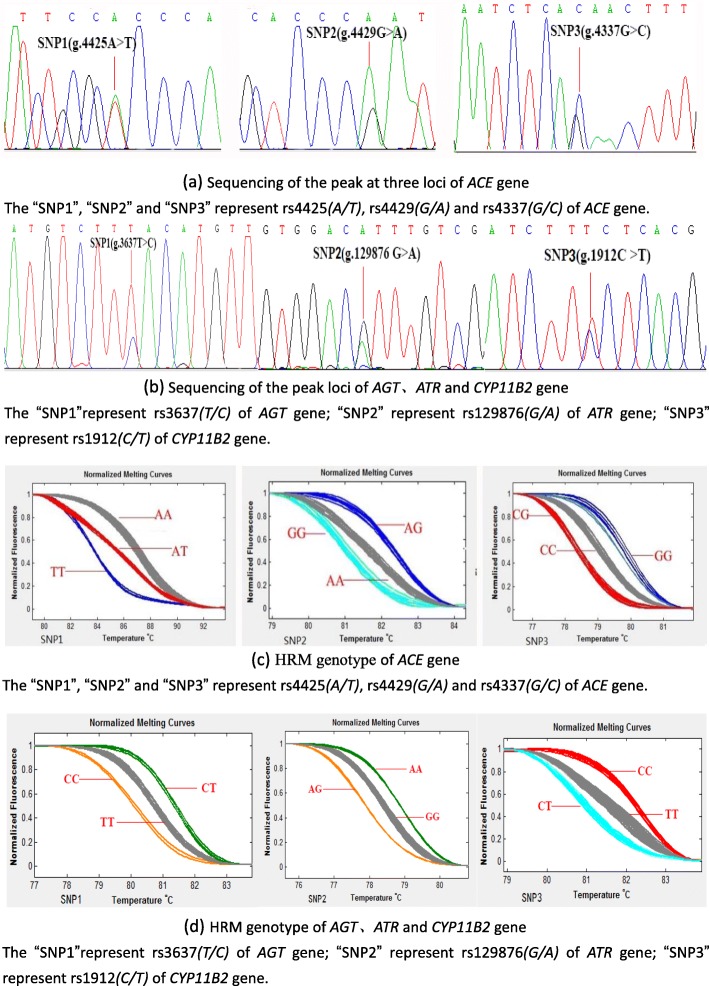
The polymorphic loci of tested genes in the studied Hui Pilgrims to *Hajj*

Polymorphism was investigated within the coding sequences of four RAAS genes: at three sites within the *ACE* gene [rs4425 (*A/T*), rs4429 (*G/A*) and rs4337 (*G/C*)] (Fig. 2a), as well as at one site each within the *AGT* gene [rs3637 (*T/C*)], *ATR* gene [rs129876 (*G/A*)] and *CYP*11B2 gene [rs1912 (*C/T*)] (Fig. 2b). High-resolution melting analysis detected three genotypes at each of the six polymorphic sites. The dominant genotypes at sites in the *ACE* gene were *AA*, *GG*, and *CC* (Fig. 2c). The dominant genotypes were *TT* at the site in the *AGT* gene; *GG* at the site in the *ATR* gene; and *TT* at the site in the *CYP11B2* gene (Fig. 2d).

**Fig. 3:**
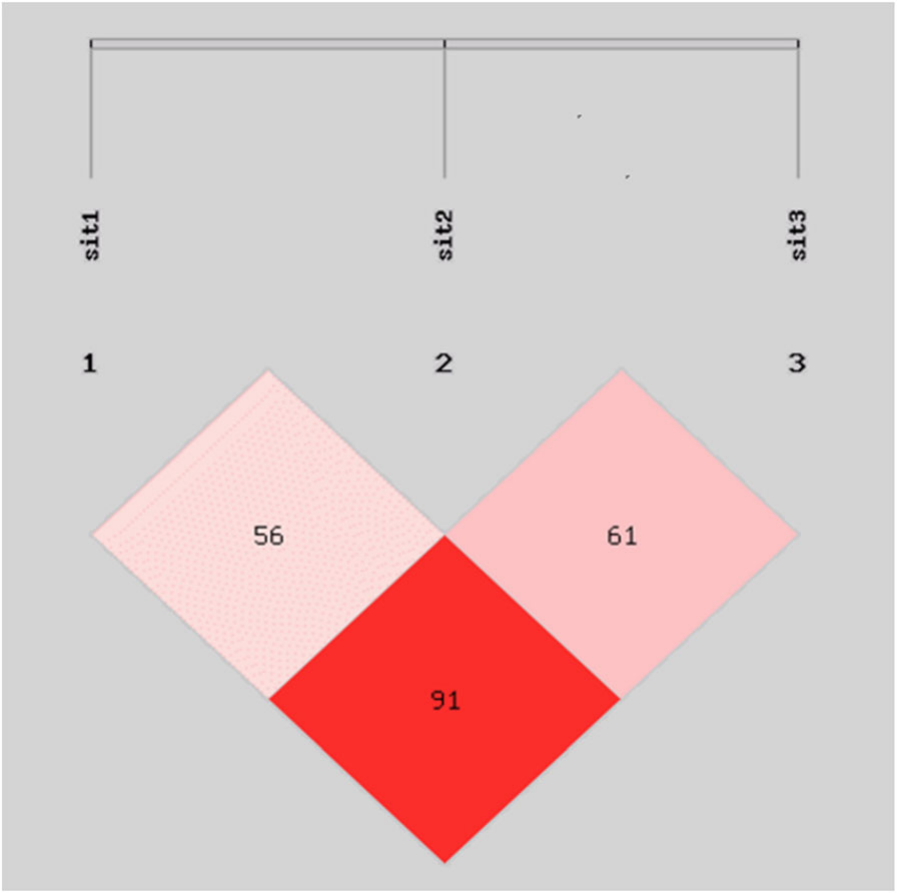
The linkage disequilibrium patterns of ACE polymorphisms

Table 3 showed the genotype at polymorphic *RAAS* sites among participants with and without hypertension. The difference of genotype frequencies among hypertension and normal groups were statistically significantat loci rs4425 (*A/T*) and rs4337 (*G/C*) in the *ACE* gene, at locus rs129876 (*G/A*) in the *ATR* gene and at locus rs1912 (*C/T*) in the *CYP*11B2 gene. Table 4 showed the homozygosity (Ho), heterozygosity (He), effective number of alleles (Ne) and polymorphism information content (PIC). PIC varied from 0.331 to 0.389 corresponding to moderate polymorphism (defined as 0.25 < PIC < 0.5). All the sites were consistent with Hardy-Weinberg equilibrium and were representative of the population based on the chi-squared test (*P > 0.05*). Figure 3 shows the results of Linkage disequilibrium calculation for ACE polymorphisms.

**Table 3:** Genotype and allele frequencies at polymorphic *RAAS* sites

Gene	Locus	Group	Genotype frequency			*P* value^*^
			AA	AT	TT	
ACE	rs4425	Normal	81 (41.5)	80 (41.3)	34 (17.4)	< 0.01
		Cases	68 (42.0)	38 (23.4)	56 (34.6)	
			GG	AG	AA	
ACE	rs4429	Normal	78 (40.0)	64 (32.8)	53 (27.2)	0.42
		Cases	76 (46.9)	47 (29.0)	39 (24.1)	
ACE	rs4337		GG	CG	CC	
		Normal	81 (41.5)	71 (36.4)	43 (22.1)	0.01
		Cases	49 (30.2)	54 (33.3)	59 (36.5)	
			GG	AG	AA	
ATR	rs129876	Normal	135 (69.2)	28 (14.4)	32 (16.4)	< 0.01
		Cases	69 (42.6)	29 (17.9)	64 (39.6)	
			TT	CT	CC	
AGT	rs3637	Normal	88 (45.1)	39 (20.0)	68 (34.9)	0.28
		Cases	79 (48.7)	22 (13.6)	61 (37.7)	
			CC	CT	TT	
CYP11B2	rs1912	Normal	67 (34.4)	31 (15.9)	97 (49.7)	< 0.01
		Cases	30 (18.5)	32 (19.8)	100 (61.7)	

*Note.* ^*^ The results from the *Chi Square Test*

**Table 4:** Genetic diversity of different mutation sites

Gene	Loci	He	Ho	Ne	PIC	*P* value^*^
ACE	rs4425	0.669	0.331	1.429	0.389	1.18
	rs4429	0.697	0.303	1.435	0.368	5.57
	rs4337	0.647	0.352	1.546	0.379	4.65
ATR	rs129876	0.846	0.154	1.182	0.370	3.60
AGT	rs3637	0.823	0.177	1.215	0.378	1.79
CYP11B2	rs1912	0.829	0.171	1.206	0.331	4.05

*Note.* He means Heterozygosity. Ho means Homozygosity. Ne means Effective allele. PIC refers to the value of a marker used to detect polymorphisms in a population. PIC depends on the number of alleles detected and their frequency distribution. The value is equal to 1 minus the sum of the squared frequencies of all alleles. PIC> 0.5 means high diversity, 0.25 < PIC< 0.5 means moderate diversity, PIC< 0.25 means low diversity. ^*^ The results from the *Chi Square Test*

**Fig. 4:**
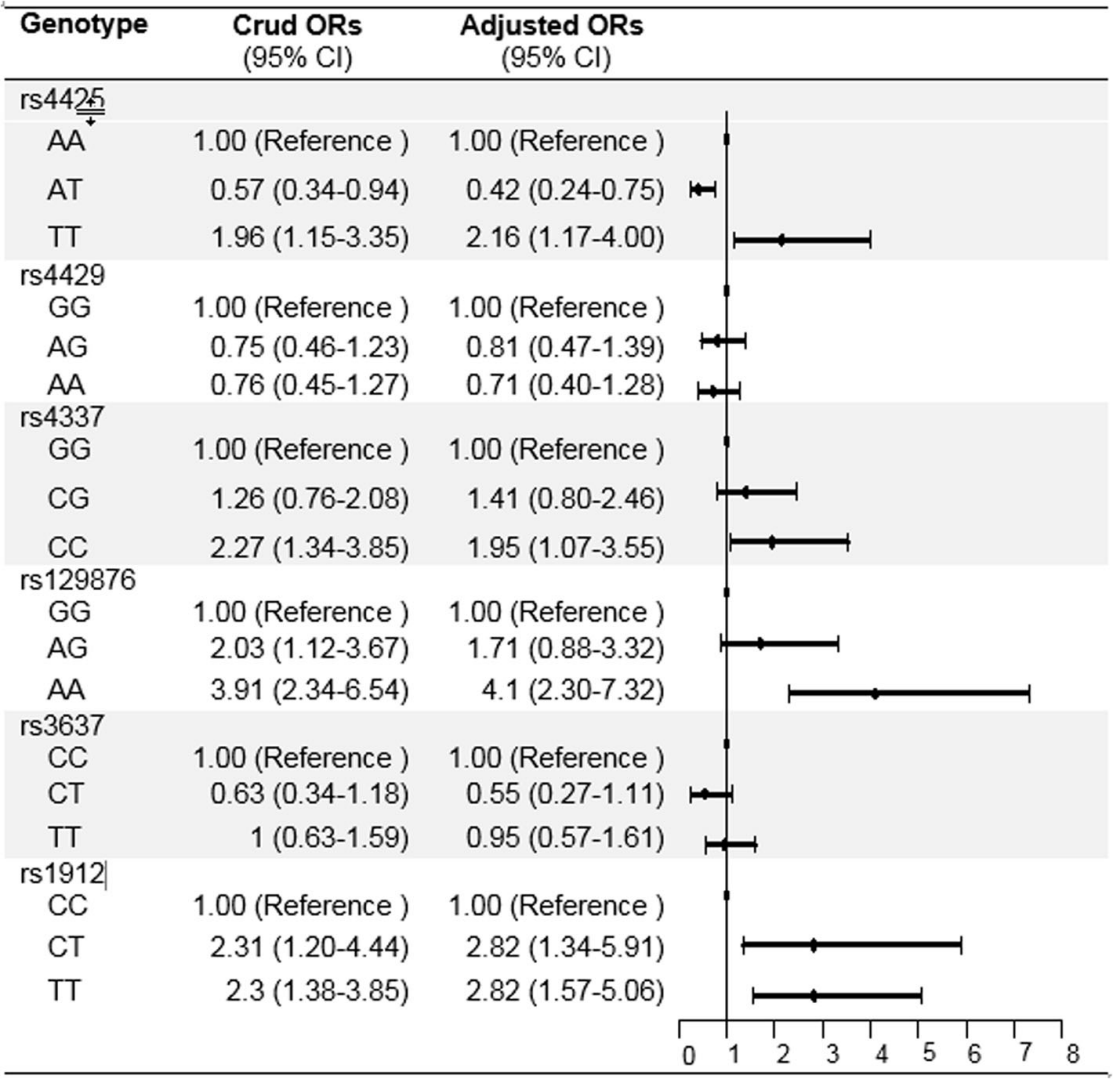
Associations between genotypes and risk of hypertension in Chinese pilgrims to *Hajj*. The dots and horizontal lines are ORs and 95% confidence intervals (CIs) using multivariate logistic regression analysis after adjustment for: age, sex, marital status, monthly family income, BMI, tobacco smoking, alcohol drinking, physical activity, diabetes and family history of hypertension

Figure 4 showed the results of multiple logistic regression models for the association between genotypes and hypertension risk in Chinese pilgrims to *Hajj*. After adjusting for traditional risk factors, including age, sex, marital status, monthly family income, BMI, tobacco smoking, alcohol drinking, physical activity, diabetes and family history of hypertension, rs4425 *TT* genotype was associated with the risk of hypertension with an adjusted OR of 2.16 (95% CI: 1.17–4.00), comparing with *AA* genotype. rs4337 *CC* genotype was associated with the risk of hypertension with an adjusted OR of 2.16 (95% CI: 1.073–3.554), comparing with *GG* genotype. Similar results were also observed for *ATR* and *CYP11B2* genotypes: compared with *ATR* rs129876 *GG* genotype, the OR of hypertension risk was 4.10 (95% CI: 2.30–7.32) for *AA* genotype. Compared with *CYP11B2* rs1912 *CC* genotype, the OR of hypertension risk was 2.82 (95% CI: 1,57–5.06) for *TT* genotype.

## Discussion

This study provides evidence that age, BMI, diabetes, preference for fried or barbecued foods and polymorphism in the *ACE, ATR*, and *CYP11B2* genes of the *RAAS* system were associated with elevated risk of hypertension among Hui *Hajj* pilgrims in Gansu Province, China. Lifestyle and genetic risks were independently associated with elevated risk of hypertension among those population.

In present study, those pilgrims are relatively older and more likely to be overweight or obese than average populations [24, 25]. What’s more, the prevalence of hypertension of the pilgrims is higher than other populations, which are consistent with previous reports [26, 27]. Therefore, the health management departments should pay more attention to chronic disease in particular hypertension among Chinese *Hajj* pilgrims. Several studies have shown a close relationship between hypertension and diabetes. The prevalence of hypertension in diabetics is about twice that in non-diabetics, what’s more striking, the prevalence peaks 10 years earlier in diabetic groups. That implicates that even individuals with the same genotype may show different phenotypic traits when exposed to different environmental factors. In our study, the prevalence of hypertension family history is similar between the hypertension and normal groups, which reflects the fact that the measurement error could not be ruled out, as the participants are older and less well educated, thus unaware or unconcerned about their parents’ and grandparents’ health.

Our results were also consistent with previous studies, which showed that population with high prevalence of hypertension was more likely to engage in poor dietary behaviors, especially eating fried or barbecued foods. Frying or barbecuing food involves a large amount of fat, and adding excess salt. Elevated cholesterol levels can directly damage the vascular endothelium and increase blood pressure. Sodium is an independent risk factor for heart disease and stroke [28]. Previous research revealed a U-shaped relationship between sodium intake and hypertension, which indicated both low and high sodium intake were associated with increased rates of hypertension [29]. The strength of the association between sodium intake and hypertension also rises with age [30, 31]. Worship frequency was the main form of daily exercise for those pilgrims, however, it is not related with the risk of hypertension in this study. Therefore, it is suggested that improving the dietary habits of Hui Muslims should be a key strategy in basic public health services and the management of basic public health services. We should adjust the allocation of resources and devote more resources to health education so as to effectively influence and improve of the dietary habits of Muslims, which will increase the control rate of hypertension while reduce the morbidity and the possibility of various complications among Hui people.

Many studies have found that more than 220 loci are closely related to hypertension, and variants of these loci can affect blood pressure levels and increase the risk of hypertension [32]. RAAS is a hormone system that regulates blood pressure and fluid balance, which plays a very important role in blood pressure regulation. In this study population, the statistically significant difference between participants with and without hypertension in the following *RAAS* sites: rs4425 and rs4337 in the *ACE* gene, rs29876 in the *ATR* gene and rs1912 in the *CYP11B2* gene. These potentially hypertension-associated polymorphisms differ from those reported in other studies on Chinese and non-Chinese populations [33, 34]. This discrepancy is consistent with a literature showing different genetic risk factors for hypertension depending on time period, geographic region and ethnicity [1, 10]. Genome-wide association studies are needed to clarify these discrepancies, which help us gain a more complete understanding of the genetic risk factors for hypertension. *ACE rs4425, ACE rs4337, ART rs129876* and *CYP11B2 rs1912* polymorphism were associated with an increased risk of hypertension in mutant homozygous genotype. It is advised that consideration be given to striving for conditions to carry out the related gene detection work and genome-wide association studies in a planned and step-by-step way among the Hui people in Gansu Province or in some key areas. The targeted and effective prevention and treatment strategies of hypertension can be adopted to reduce the economic cost of related diseases and improve the output/input ratio. Moving toward precision medicine will have positive sociological and health economics implications.

This finding reflected the potential positive relationship between the *RAAS* system and salt-sensitive hypertension [35, 36]. For example, expression of *ATR* can increase renal transport or vascular smooth muscle cell reactivity, while *ACE* helps metabolize vasoactive peptides. *AGT* is associated with a reduction in blood pressure after salt intake [3, 37]. Salt intake may affect the *RAAS* system to a greater extent in older people, whose blood pressure is extremely sensitive to salt intake [38]. Behavior often determines whether or not people are exposed to environmental risk factors and the degree of exposure. Therefore, the interaction between genes and environment based on behavior research is conducive to further clarifying the pathogenesis of hypertension, and the intervention of behavioral factors with high variability is easy to be realized, providing ideas for the effective prevention and treatment of hypertension. While the cross-sectional nature of this study prevents us from exploring causal relationships between behavioral and genetic factors and hypertension, our results provide testable hypotheses for future prospective studies.

Several strengths and limitations need to be kept in mind when interpreting these findings. First, studies on lifestyle and genetic risks in Chinese pilgrims are very limited, we comprehensively examined the factors associated with hypertension and their interactions with potential genetic factors among Hui Hajj pilgrims in China. Second, comprehensive information regarding potential confounders was also carefully measured and analyzed, thus minimizing the probability of bias. Third, we further evaluated the interactions between lifestyle and genetic factors with risk of hypertension. The main limitation of this study is its cross-sectional nature, causality cannot be established. Another limitation is some common risk factors for hypertension such as raised non-HDL cholesterol and chronic kidney disease were not included in this study. Additionally, we did not to distinguish participants who are newly diagnosed with their clinical examination, and those who are known to have a clinically diagnosed hypertension prior to their participation in this study that would lead to a biased conclusion.

## Conclusion

In summary, this study observed that lifestyle and genetic risks were independently associated with hypertension risk *Hui* pilgrims in China. Identifying individuals who are at high risk of hypertension is helpful in providing them an efficient means of prevention, further longitudinal studies and serum levels of components testing are warranted to replicate this finding.

## Additional file

Additional file 1: Supplemental Tables. (DOCX 19 kb)

## Data Availability

Original data of this study will not be shared because it is part of health examination data and blood samples of the Gansu International Travel Healthcare Center.

## Abbreviations

ACE: Angiotensin converting enzyme
AGT: Angiotensinogen
Ang: Angiotensin
ATR: Angiotensin II receptor
BMI: Body mass index
CVD: Cardiovascular disease
CYP11B2: Aldosterone synthase
DBP: Diastolic blood pressure
DNA: Deoxyribonucleic acid
FPG: Fasting plasma glucose
He: Heterozygosity
Ho: Homozygosity
Ne: Effective number of allele gene
PCR: Polymerase Chain Reaction
PIC: Polymorphism Information Content
RAAS: Renin-angiotensin-aldosterone system
SBP: Systolic blood pressure
WHO: World Health Organization
