# Supplementary material for: A cross-sectional study on factors associated with hypertension and genetic polymorphisms of renin-angiotensin-aldosterone system in Chinese hui pilgrims to hajj
Source: BMC Public Health. 2019 Sep 4;19:1223. doi: 10.1186/s12889-019-7357-1 (PMC6727391; doi:10.1186/s12889-019-7357-1)
Supplement: Supplementary file 1 — Supplemental Tables. (DOCX 19 kb) [file 12889_2019_7357_MOESM1_ESM.docx]

***Supplemental Table 1*** Primers of CDS range of blood ATG、ACE、ATR and CYP11B2

| Primers | Primers sequences（5′-3′） | Tm（℃） | Length（bp） | Locus |
| --- | --- | --- | --- | --- |
| ATG | F:CTCTATCCTTGGCTTGT | 48 | 706 | 3250-3955 |
|  | R:ATTGGAGCAGGTATGAA |  |  |  |
| ACE | F:GCAACAACGCACTTTCAC | 54 | 582 | 4329-4911 |
|  | R:CCTTCTTCCTCCATTCCA |  |  |  |
| ATR | F:CTGGGTTTGGTTCACG | 52 | 821 | 128384-129105 |
|  | R:ACGGCAGTCCTGTCACTCTAT |  |  |  |
| CYP11B2 | F:GGAAGGGAACGCTATTGG | 55 | 423 | 1507-1929 |
|  | R:GACAGTGATGACAGGCAGAA |  |  |  |

***Supplemental Table 2*** Information of primer sequences for genotyping mutations by HRM

| Loci | Primers sequences（5′-3′） | Tm（℃） | Length（bp） |
| --- | --- | --- | --- |
| ATG3637（T/C） | F:TTCCTGTGCATGTCTTTAC | 49 | 90 |
|  | R:CCTTTATCCAGTATGACCG |  |  |
| ACE4425（A/T） | F:GGTCGGGATGTGATTC | 52 | 85 |
|  | R:TCGCATTGGGTGGATA |  |  |
| ACE4429（G/A） | F:CCTGGTGACGGAATAAA | 52 | 153 |
|  | R:CCATGCAACCCACTCTA |  |  |
| ACE4337（G/C） | F:TCTTTCTACCTCCCAATC | 50 | 78 |
|  | R:GAAGTACATGCCCTGCT |  |  |
| ATR129876（G/A） | F:TCGTCGTGTCTGACTCTT | 50 | 129 |
|  | R:CGGTTTGATTCCTGGGT |  |  |
| CYP11B2  1912（C/T） | F:CCTCCCTCAAAGCAAAT | 50 | 85 |
|  | R:TTCCCTGGTTGGATGAT |  |  |

***Supplemental Table 3*** Information of low and high calibrator sequence

| High and low calibrator | Calibrator（5′-3′） | Tm（℃） |
| --- | --- | --- |
| Low up | GTATTATATTTATATATATATAATTAATA  TTATAAATATTTTATAATTTAA-C3-3' | 57 |
| Low DN | TTAAATTATAAAATATTTATAATATTAATTA  TATATATATAAATATAATAC-C3-3' | 53 |
| High up | GCCCGCCCCTCCGCTTCCGCACCTCCAGCAGCCGCT  CAGAGTCTCGGGTCAGTGCCGGCCGCGC-C3-3' | 86 |
| High DN | GCGCGGCCGGCACTGACCCGAGACTCTGAGCGGCTG  CTGGAGGTGCGGAAGCGGAGGGGCGGGC-C3-3' | 87 |
